# Predictive value of T cell receptor repertoire profiling for immunosuppressive therapy in severe aplastic anemia

**DOI:** 10.1016/j.gendis.2023.03.027

**Published:** 2023-04-29

**Authors:** Cunte Chen, Yuling Zhang, Dongpei Lu, Zelong Zhang, Jun Yang, Xiaowei Chen, Ming Zhou, Wenjian Mo, Caixia Wang, Qinghua Cai, Yumiao Li, Ruiqing Zhou, Shilin Xu, Wei Zhou, Tingfen Deng, Shiyi Pan, Yanli Xu, Shunqing Wang, Yuping Zhang

**Affiliations:** a Department of Hematology, Guangzhou First People's Hospital, South China University of Technology, Guangzhou, Guangdong 510180, China; b Guangzhou Junruikang Biotechnology Co., Ltd, Guangzhou, Guangdong 510700, China

Increasing evidence supports the hypothesis of autologous immune attack in severe aplastic anemia (SAA): the predominant role of activated cytotoxic T cells (CTL) expressing γ-interferon in inhibiting the growth of bone marrow (BM) cells, putative autoantigens, and oligoclonal expansion of CD8^+^ T cells.^1^ For SAA patients, the definitive therapies are immunosuppressive therapy (IST) or hematopoietic stem transplantation (HSCT); IST is most widely applied in the clinic because of the lack of HLA-matched sibling or unrelated donors, patients' age, and the cost of HSCT.^2^^,^^3^ However, only about 60% of SAA patients are responders after receiving IST, and less than 10% achieve complete remission (CR)^2^^,^^3^; effective biomarkers for the efficacy prediction of IST in SAA patients are lacking.^3^ Our previous publications have demonstrated that T cell receptor (TCR) repertoire profiling has been identified as a biomarker for predicting the clinical outcomes and efficacy of patients.^4^^,^^5^ However, systematic evaluation of the predictive value of the TCR repertoire for SAA patients during IST is still little known.

TCRβ chain (TCRβ) sequencing was used to characterize the TCR repertoires of newly diagnosed SAA patients from the GSE101660 dataset and our clinical center (GZFPH), and patients receiving IST for 1, 3, 6, and 12 months in the GZFPH dataset (Fig. S1). TCR rearrangement with a frequency greater than 0.01% was defined as a TCR clone and the amino acid length of CDR3 in TCR clones was first explored. The peak CDR3 length of CD8^+^ and CD4^+^ T cells was 13–15 in both healthy individuals (HIs) and SAA patients in the GSE101660 dataset, which was also shown in SAA in our clinical center dataset (GZFPH) (Fig. S2A, B). The frequency of TCR clones in CD8^+^ T cells of SAA patients was significantly higher than HIs, while a lower frequency of TCR clones was shown in CD4^+^ T cells of SAA patients in the GSE101660 dataset (*P* < 0.001, Fig. S2C). As expected, the frequency of TCR clones in CD8^+^ T cells of SAA patients significantly increased compared with CD4^+^ T cells (*P* < 0.001). This result was confirmed in the number of TCR clones in CD8^+^ T cells (*P* = 0.015, Fig. S2D). Although TCR repertoire diversity was not statistically significant between HIs and SAA (*P* = 0.350), the TCR repertoire diversity of CD8^+^ T cells was lower than that of CD4^+^ T cells in the GSE101660 dataset of the TCRdb database (*P* < 0.001, Fig. S2E), which might be due to the increased proportion of CD8^+^ CD4^+^ T cells in SAA patients and the enhanced function of CD8^+^ T cells, leading to the oligoclonal expansion of TCR rearrangements. Interestingly, compared with the newly diagnosed SAA patients, the TCR diversity of patients after receiving IST for 1, 3, 6, and 12 months decreased in the GZFPH dataset (*P* < 0.05, Fig. S2F). To further evaluate TCR rearrangements associated with SAA patients, the differential frequency of V-J usage was first analyzed. There were 6 frequently used and 11 less used TCR rearrangements identified in CD8^+^ T cells between HIs and SAA patients in the GSE101660 dataset, and 10 frequently used and 1 less used TCR rearrangements were identified in CD4^+^ T cells between HIs and SAA patients (Fig. S3A, B). Moreover, a total of 36 frequently used and 3 less used TCR rearrangements were identified comparing CD8^+^ and CD4^+^ T cells in SAA patients in the GSE101660 dataset (Fig. S3C). Taken together, a total of 65 overlapped frequently and less used TCR rearrangements were used for the following analysis.

To identify the TCR rearrangements related to the efficacy of IST, the efficacy rate of IST was first analyzed. The response rate of SAA patients to IST was 66.7% in the GZFPH dataset (Fig. S4A, B). Then, 65 differentially expressed TCRs obtained from the GSE101660 dataset were further used for the analysis between newly diagnosed patients and patients receiving IST for 12 months. Notably, 3 Vβ6-5 and 5 Vβ20-1 decreased after SAA patients receiving IST for 12 months in the partial remission (PR)/CR group (*P* < 0.05, Fig. S4C). However, these 8 TCR rearrangements were not statistically significant after SAA patients receiving IST for 1, 3, and 6 months compared with newly diagnosed patients in the no remission (NR) group (*P* > 0.05, Fig. S4D). To evaluate the sensitivity and accuracy of these 8 TCRs in the efficacy prediction of these 8 TCRs in SAA patients' response to IST, we performed a ROC curve analysis in the GZFPH dataset. There was a clear trend suggesting that high frequency of Vβ20-1 Jβ1-5, Vβ20-1 Jβ1-2, and Vβ20-1 Jβ1-1 was positively correlated with PR/CR of patients receiving IST (AUC ≥0.88, Fig. 1A). However, only Vβ20-1 Jβ1-5 was the best model in predicting the efficacy of IST, which was internally validated by 100 repeated 10-fold cross-validation (Fig. 1B). Importantly, the high frequency of Vβ20-1 Jβ1-5 had a very high accuracy in predicting PR/CR of SAA patients' response to IST (AUC = 100%; *P* = 0.064) (Fig. 1C). We further obtained the optimal cut-point 0.00826 in the ROC, indicating that its sensitivity in predicting PR/CR of SAA patients' response to IST was as high as 100% when the frequency of Vβ20-1 Jβ1-5 was greater than 0.00826, which was confirmed in the clinical utility curve (Fig. 1C, D). Moreover, the high frequency of Vβ20-1 Jβ1-5 was significantly associated with favorable event-free survival (EFS) for SAA patients (*P* = 0.018, Fig. 1E). Interestingly, the frequency of Vβ20-1 Jβ1-5 in the PR/CR group was higher than that in the NR group, though there was no statistical significance at that point (*P* = 0.069, Fig. 1F). In addition, Vβ20-1 Jβ1-5 was decreased after SAA patients receiving IST for 1, 3, 6, and 12 months in the PR/CR group (*P* = 0.006), other than the NR group (*P* = 0.594) (Fig. 1G; Fig. S5). TCR expressions can be regulated during lymphocyte development and activation events, and Vβ20-1 Jβ1-5 was significantly up-regulated in CD8^+^ T cells compared with CD4^+^ T cells (Fig. S2C). Therefore, correlations with the up-regulated CD8^+^ T cells were evaluated, which would relatively exclude the effects of T-cell counts on the frequency of Vβ20-1 Jβ1-5 (Fig. S6A). The frequency of Vβ20-1 Jβ1-5 was normalized to that of CD8^+^ T cells, which was significantly up-regulated in the PR/CR group compared with the NR group (*P* = 0.044, Fig. S6B). To identify the clonotype contribution of Vβ20-1 Jβ1-5, we further explored the amino acid and nucleotide sequences. The results demonstrated that the amino acid and nucleotide at both ends of the CDR3 region were almost completely conserved, and the middle sequences were highly diverse (Fig. S6C, D). Taken together, SAA patients might benefit from IST when the frequency of Vβ20-1 Jβ1-5 was greater than 0.00826 in newly diagnosed patients.

**Figure 1:**
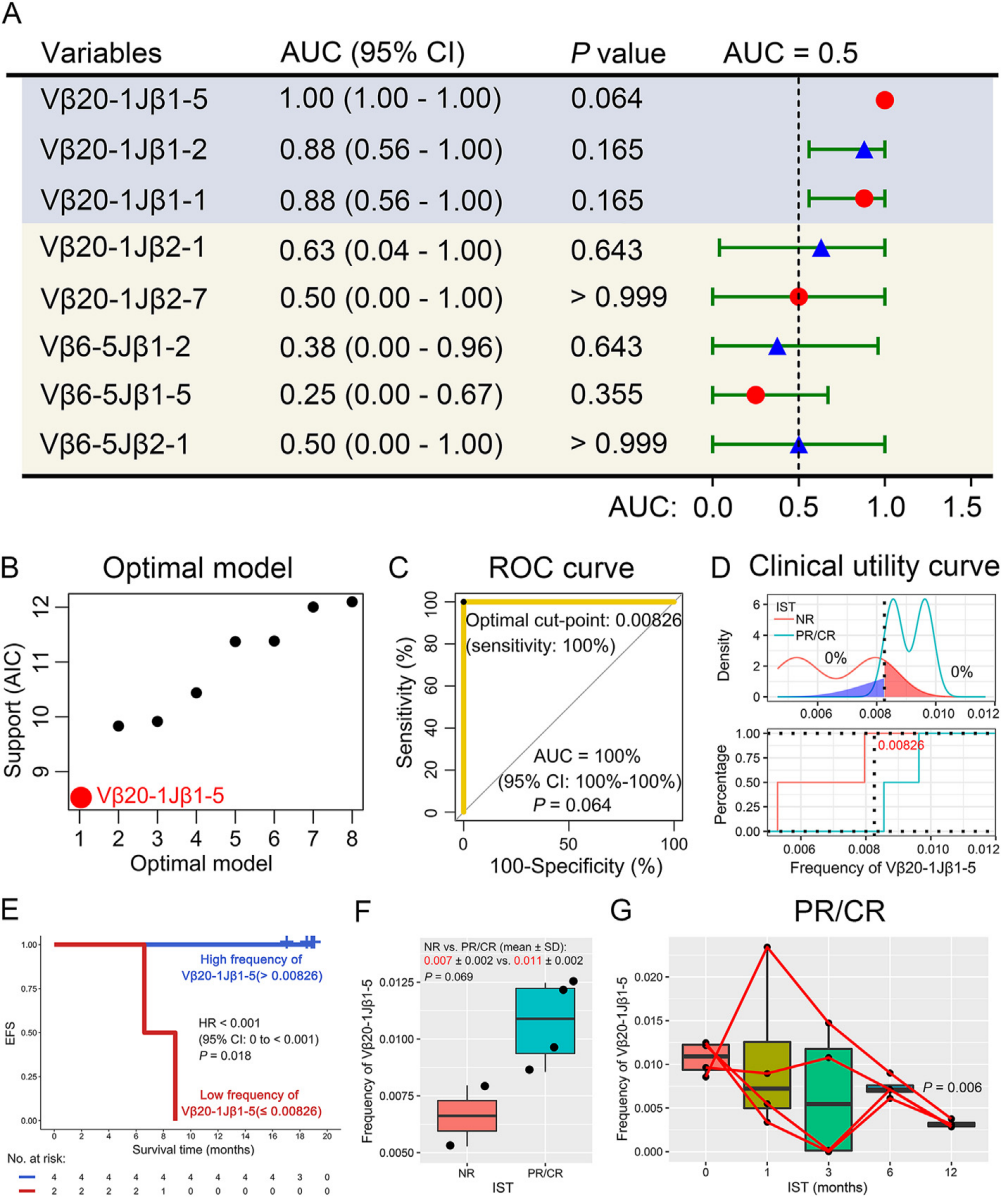
Vβ20-1 Jβ1-5 was associated with the clinical outcomes of SAA patients in the GZFPH dataset. **(A)** The area under the receiver operating characteristic curve (ROC) was used to evaluate the efficacy prediction of immunosuppressive therapy (IST). **(B)** Akaike information criterion (AIC) profile of the best to the worst model. **(C)** The sensitivity and accuracy of frequently used Vβ20-1 Jβ1-5 in predicting the response to IST in SAA patients. **(D)** Clinical utility curve for predicting the response to IST. **(E)** The event-free survival (EFS) for the low and high frequency of Vβ20-1 Jβ1-5 subgroups in SAA patients. **(F, G)** The difference of Vβ20-1 Jβ1-5 frequency between no remission (NR) versus partial remission (PR)/complete remission (CR) subgroups (F), and SAA patients receiving IST for 0, 1, 3, 6, and 12 months in the PR/CR subgroup (G).

In conclusion, we for the first time described that a high frequency of Vβ20-1 Jβ1-5 was associated with favorable clinical outcomes and efficacy in SAA patients receiving IST, which might be a biomarker to guide IST for SAA patients.

## Author contributions

YPZ and SQW contributed to the concept development and study design, coordinated the research, and helped write the manuscript. CTC collected the clinical information, analyzed the data, and wrote the manuscript. YLZ, YLX, and QHC performed the experiments. XWC, MZ, WJM, and CXW diagnosed and treated the patients and provided clinical samples. YML, RQZ, SLX, WZ, and TFD collected the clinical samples. SYP contributed to the follow-up of SAA patients. DPL, ZLZ, and JY performed TRBV deep sequencing and bioinformatics analysis. All authors read and approved the final manuscript.

## Ethics declaration

This study was approved by the Ethics Committee of Guangzhou First People's Hospital. All participants provided written informed consent.

## Conflict of interests

The authors declare that they have no competing interests.

## Funding

This study was supported by grants from the Innovative Clinical Technique of Guangzhou, China (No. 2019GX04 and 2023C-GX01 to YPZ and SQW, respectively), 2019 Annual Research Project of The China Marrow Donor Program (No. CMDP201902 to SQW), Guangzhou Municipal Science and Technology project (China) (No. 202002030035 to SQW), and the Natural Science Foundation of Guangdong Province, China (No. 2018A0303130179 to MZ).

## Data availability

The datasets used and analyzed in the current study are available from the corresponding author upon reasonable request.

## References

[1] Giudice V., Feng X., Lin Z. (2018). Deep sequencing and flow cytometric characterization of expanded effector memory CD8^+^ CD57^+^ T cells frequently reveals T-cell receptor Vβ oligoclonality and CDR3 homology in acquired aplastic anemia. Haematologica.

[2] Hu J., Zhang L., Zhao X. (2022). First-line immunosuppressive therapy with rATG and CsA for severe aplastic anemia: 15 years' experience. Ann Hematol.

[3] Patel B.A., Townsley D.M., Scheinberg P. (2022). Immunosuppressive therapy in severe aplastic anemia. Semin Hematol.

[4] Chen C., Liu S.Y.M., Chen Y. (2022). Predictive value of TCR Vβ-Jβ profile for adjuvant gefitinib in EGFR mutant NSCLC from ADJUVANT-CTONG 1104 trial. JCI Insight.

[5] Chen C., Liu S.M., Chen Y. (2022). Poor prognosis of intra-tumoural TRBV6-6 variants in EGFR-mutant NSCLC: results from the ADJUVANT-CTONG1104 trial. Clin Transl Med.

