# Supplementary material for: Predictive value of T cell receptor repertoire profiling for immunosuppressive therapy in severe aplastic anemia
Source: Genes Dis. 2023 Apr 29;11(1):95–8. doi: 10.1016/j.gendis.2023.03.027 (PMC10425835; doi:10.1016/j.gendis.2023.03.027)
Supplement: Multimedia component 1 [file mmc1.docx]

**Predictive value of T cell receptor repertoire profiling for immunosuppressive therapy in severe aplastic anemia**

**Running title:** Predictive value of TCR in SAA

**Methods and materials**

**SAA patients' samples**

During the period from March 1, 2021, to November 30, 2021, a total of 6 SAA patients’ samples including 28 peripheral blood (PB) samples from the newly diagnosed patients and patients receiving IST for 1, 3, 6, and 12 months were collected at Guangzhou First People's Hospital (GZFPH). All SAA patients in this study met the following inclusion criteria: (1) Acquired SAA was diagnosed according to the modified Camitta criteria ^1, 2^; (2) Voluntarily underwent IST as the ﬁrst-line treatment; (3) Patients who have not been treated with CsA or HSCT before IST. For IST regimens, SAA patients received rabbit ATG (5 mg/Kg/d from day -5 to day -1) plus CsA (3-6 mg/Kg/d from day -5 to day -1), maintained the concentration of CsA at 150-250 ng/mL. The detailed criteria for response to IST in SAA, involving partial remission (PR), CR, and not remission (NR), were described by Marsh *et al* ^1, 2^. Clinical information of 6 SAA patients, including age, gender, survival time, event, and Eastern Cooperative Oncology Group (ECOG) score, was listed in Table S1. The event-free survival (EFS) was deﬁned as the time from the date of diagnosis to the date of death or endpoint of follow-up. The follow-up for SAA patients was ended when patients were evaluated as NR after receiving IST for 6 months, otherwise, the follow-up was ongoing, and the last follow-up time was on December 1, 2022. This study was approved by the Ethics Committee of Guangzhou First People's Hospital. All participants were provided with written informed consent.

**GSE101660 dataset**

The TCR repertoire sequencing data of sorted CD4+ and CD8+ T cells from the PB samples of 12 SAA patients and 9 healthy individuals (HIs) in the GSE101660 dataset was downloaded from the Gene Expression Omnibus (GEO) database (<https://www.ncbi.nlm.nih.gov/geo/>) ^3^. The TCR repertoire diversity of CD4+ and CD8+ T cells from SAA patients and HIs in the GSE101660 dataset was obtained from the TCRdb database (<http://bioinfo.life.hust.edu.cn/TCRdb/>) ^4^. The clinical characteristics were shown in Table S1. Since the GSE101660 dataset was publicly available, no local ethics committee approval was required.

**TRBV deep sequencing**

PB samples of SAA patients were collected for TCR repertoire sequencing in Guangzhou Junruikang Biotechnology Co., Ltd. PB samples were used for RNA extraction and polymerase chain reaction (PCR) to construct TCR libraries according to the manufacturer’s instructions. TCR libraries were then sequenced using the Illumina HiSeq platform. For TCR profiling, fastq files were first processed to remove adapters and low-quality reads with trimmomatic and Non-V-J paired reads were further removed. Clean reads were subsequently assembled using MiXCR. Reads were aligned to reference V or J gene segments according to the ImMunoGeneTics (IMGT) database. Finally, amino acid sequences, nucleotide sequences, counts and frequency of V-J genes, CDR3 length, and Shannon index were analyzed.

**Flow cytometry**

The antibodies including CD3-APC (clone HIT3a) and CD8-APC-H7 (clone SK1) were purchased from Biolegend (San Diego, USA). PB samples were lysed by erythrocyte lysate for 10 - 15 min. Then, cells were washed with 1 x phosphate-buffered saline (PBS) and stained with anti-CD3 and anti-CD8 for 20 min at room temperature in dark. Cells were washed with 1 x PBS, and the CD3 and CD8 molecule distribution were detected using a BD FACSVerse multicolor ﬂow cytometer (BD Biosciences, San Jose, USA) ^5^.

**Statistical Analysis**

All statistical analyses were conducted by R software (version 4.2.1). Two subgroups of quantitative variables were compared by the Mann-Whitney-Wilcoxon and paired t-test, as appropriate, while multiple subgroups of quantitative variables were compared by the Kruskal-Wallis test ^6-8^. The area under curve (AUC) in the receiver operating characteristic curve (ROC) and the optimal cut-point of AUC was determined by the package "pROC" ^9^. The best model was obtained by the package "glmulti" ^10^. Differences in subgroups in Kaplan-Meier curves were compared using the log-rank test in the R package "survival" ^11-13^. The clinical utility curve was plotted using the R packages "ggpubr", " data.table", "pROC", and "eoffice" ^9^. The consensus of nucleotide and amino acid sequences was aligned and calculated by the package "msa" ^10, 14, 15^. A two-tailed *P* < 0.05 was considered statistically significant.

**References**

1. Killick SB, Bown N, Cavenagh J, et al. Guidelines for the diagnosis and management of adult aplastic anaemia. *Br J Haematol*. 2016; 172(2):187-207.

2. Marsh JC, Ball SE, Cavenagh J, et al. Guidelines for the diagnosis and management of aplastic anaemia. *Br J Haematol*. 2009; 147(1):43-70.

3. Giudice V, Feng X, Lin Z, et al. Deep sequencing and flow cytometric characterization of expanded effector memory CD8(+)CD57(+) T cells frequently reveals T-cell receptor Vβ oligoclonality and CDR3 homology in acquired aplastic anemia. *Haematologica*. 2018; 103(5):759-769.

4. Chen SY, Yue T, Lei Q, Guo AY. TCRdb: a comprehensive database for T-cell receptor sequences with powerful search function. *Nucleic Acids Res*. 2021; 49(D1):D468-d474.

5. Huang G, Zhang Y, Wei X, et al. CD8(+)GITR(+) T cells may negatively regulate T cell overactivation in aplastic anemia. *Immunol Invest*. 2021; 50(4):406-415.

6. Chen C, Nie D, Huang Y, et al. Anticancer effects of disulfiram in T-cell malignancies through NPL4-mediated ubiquitin-proteasome pathway. *J Leukoc Biol*. 2022.

7. Li K, Chen C, Gao R, et al. Inhibition of BCL11B induces downregulation of PTK7 and results in growth retardation and apoptosis in T-cell acute lymphoblastic leukemia. *Biomark Res*. 2021; 9(1):17.

8. Huang X, Chen C, Zhong M, et al. Lower BCL11B expression is associated with adverse clinical outcome for patients with myelodysplastic syndrome. *Biomark Res*. 2021; 9(1):46.

9. Chen C, Chen S, Luo G, et al. High expression of TMEM244 is associated with poor overall survival of patients with T-cell lymphoma. *Biomark Res*. 2022; 10(1):46.

10. Chen C, Liu SM, Chen Y, et al. Poor prognosis of intra-tumoural TRBV6-6 variants in EGFR-mutant NSCLC: Results from the ADJUVANT-CTONG1104 trial. *Clin Transl Med*. 2022; 12(4):e775.

11. Chen C, Liang C, Wang S, et al. Expression patterns of immune checkpoints in acute myeloid leukemia. *J Hematol Oncol*. 2020; 13(1):28.

12. Wang P, Chen Y, Long Q, et al. Increased coexpression of PD-L1 and TIM3/TIGIT is associated with poor overall survival of patients with esophageal squamous cell carcinoma. *J Immunother Cancer*. 2021; 9(10).

13. Chen C, Liu S, Jiang X, et al. Tumor mutation burden estimated by a 69-gene-panel is associated with overall survival in patients with diffuse large B-cell lymphoma. *Exp Hematol Oncol*. 2021; 10(1):20.

14. Chen C, Liu SM, Chen Y, et al. Identification of TCR rearrangements specific for genetic alterations in EGFR-mutated non-small cell lung cancer: results from the ADJUVANT-CTONG1104 trial. *Cancer Immunol Immunother*. 2022.

15. Chen C, Liu SM, Chen Y, et al. Predictive value of TCR Vβ-Jβ profile for adjuvant gefitinib in EGFR mutant NSCLC from ADJUVANT-CTONG 1104 trial. *JCI insight*. 2022; 7(1).

**Figure legends:**


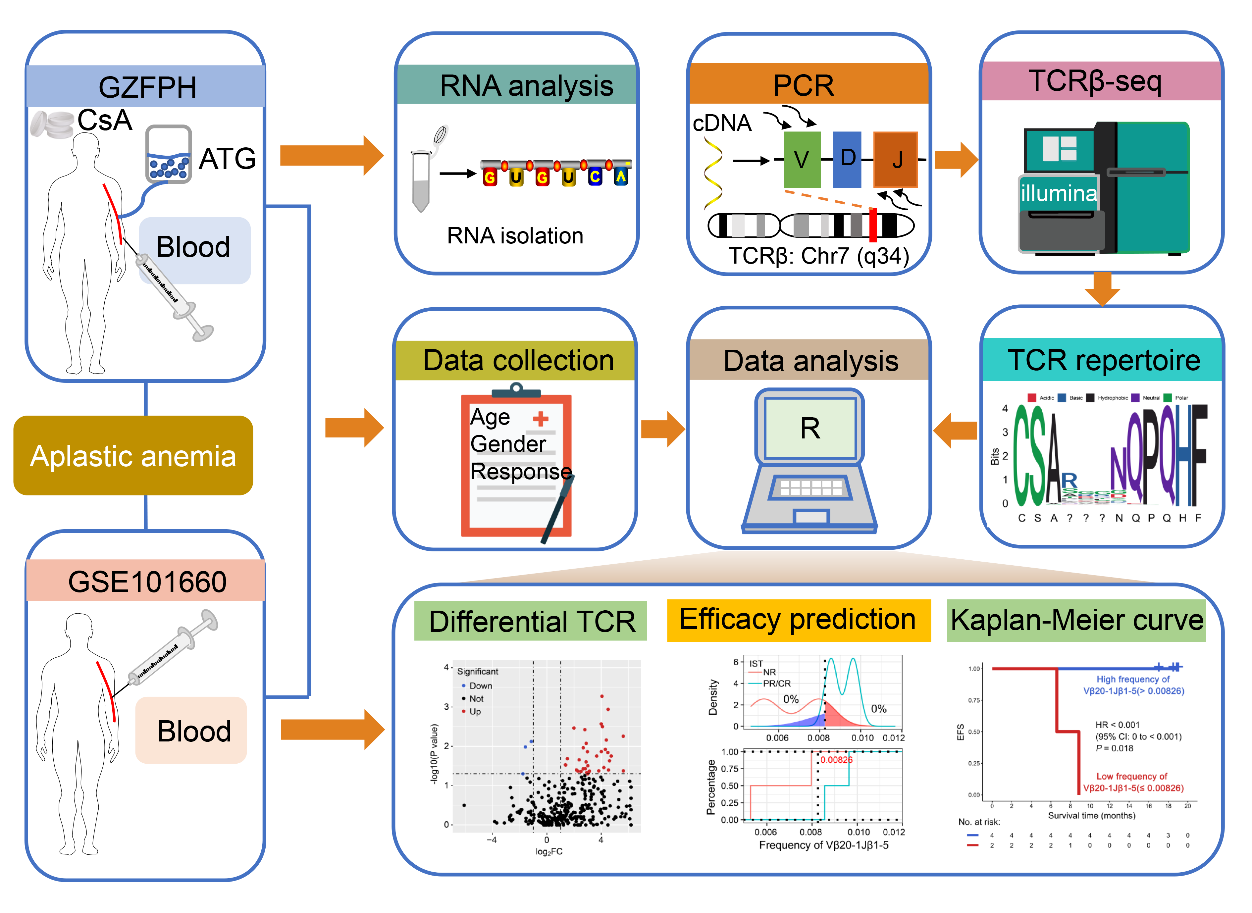


**Figure S1.** Study schematics. Peripheral blood from 6 patients with severe aplastic anemia (SAA) who were newly diagnosed and treated with anti-human thymocyte immunoglobulin (ATG) and cyclosporine A (CsA) for 1, 3, 6, and 12 months were collected from Guangzhou First People's Hospital (GZFPH) for ribonucleic acid (RNA) analysis, polymerase chain reaction (PCR) and T cell receptor β chain (TCRβ) sequencing. Then, the relationship between TCR repertoire and clinical characteristics was analyzed by bioinformatics. Finally, the publicly available dataset (GSE101660) and the GZFPH dataset were used for differential TCR, efficacy prediction, and Kaplan-Meier curve analysis.


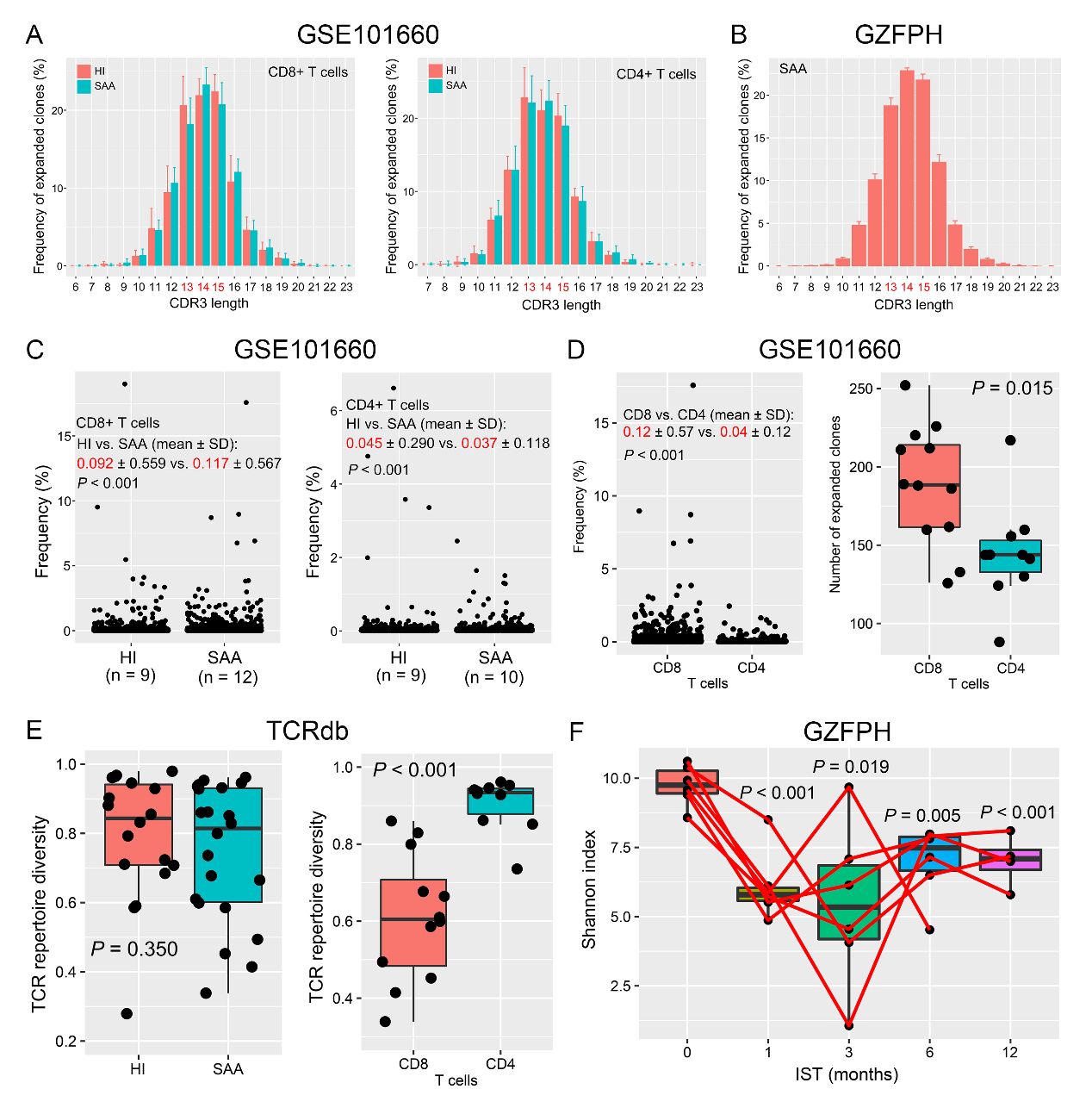


**Figure S2.** Analysis of complementarity determining region 3 (CDR3) length and TCR repertoire diversity in SAA. A-B: CDR3 length in the GSE101660 (A) and GZFPH (B) datasets. (C) The clonal TCR frequency of CD8+ (left panel) and CD4+ T cells (right panel) between healthy individual (HI) and SAA patients in the GSE101660 dataset. (D) The frequency (left panel) and number (right panel) of frequently used TCRs between CD8+ and CD4+ T cells in SAA patients were compared in the GSE101660 dataset. (E) The differences of TCR repertoire diversity in the HI vs. SAA (left panel) and CD8+ vs. CD4+ T cells (right panel) in the GSE101660 of the TCRdb database. (F) The TCR repertoire diversity (Shannon index) of SAA patients receiving IST for 0, 1, 3, 6, and 12 months in the GZFPH dataset.


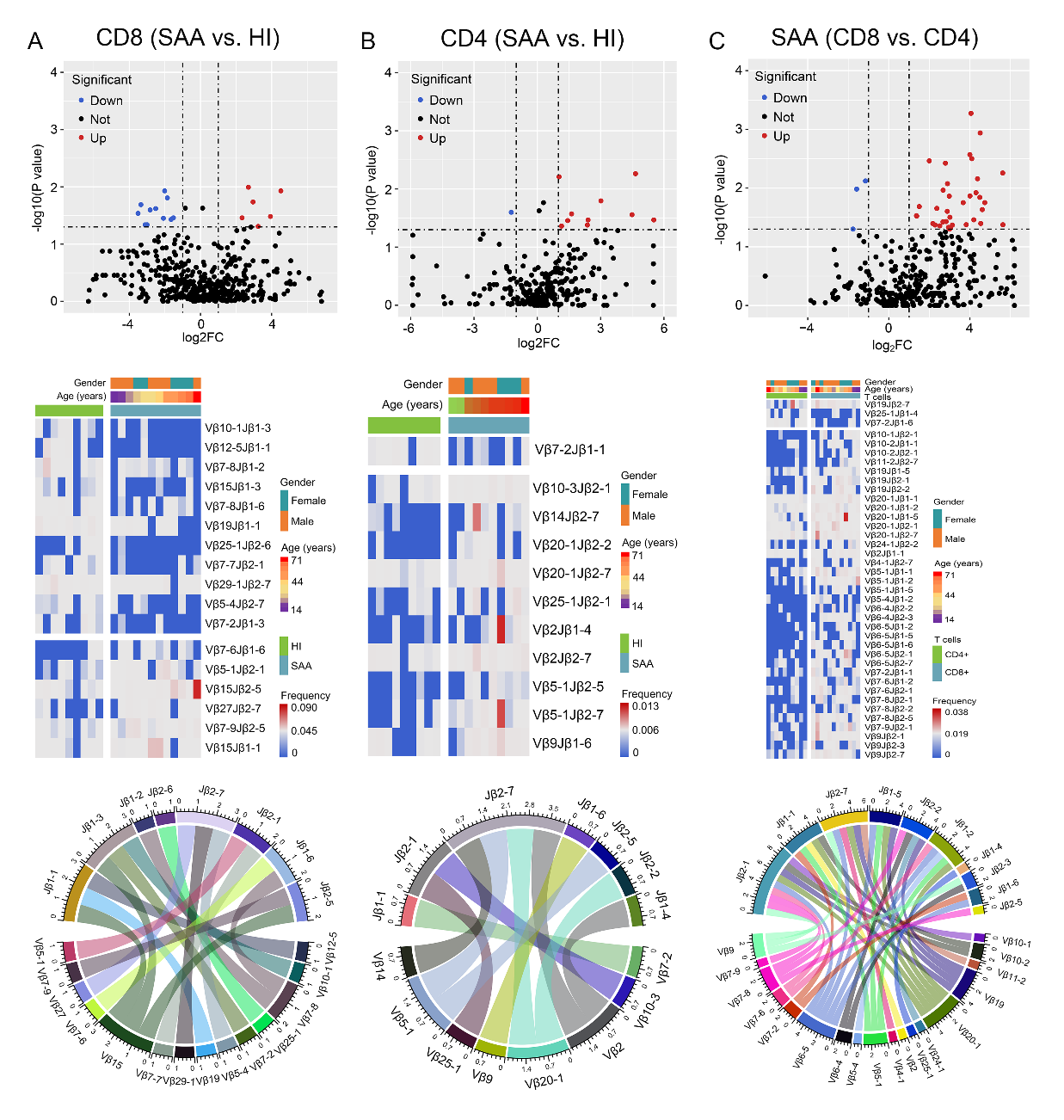


**Figure S3.** A-C: Identification of differentially expressed TCR rearrangements based on CD8+ T cells (SAA vs. HI) (A), CD4+ T cells (SAA vs. HI) (B), and SAA (CD8+ vs. CD4+ T cells) (C) in the GSE101660 dataset. Volcano plots show the differentially expressed TCRs between two subgroups (upper panel). An absolute value of the fold change (FC) > 2 and *P* < 0.05 were used as a threshold to identify the differentially expressed TCRs. The red, blue, and black dots represent upregulated, downregulated, and not statistically significant TCRs, respectively. The heatmap plot shows the frequency distribution of the differentially expressed TCRs between two subgroups (middle panel). The V-J usages of differentially expressed TCRs were described by the chord plots (bottom panel).


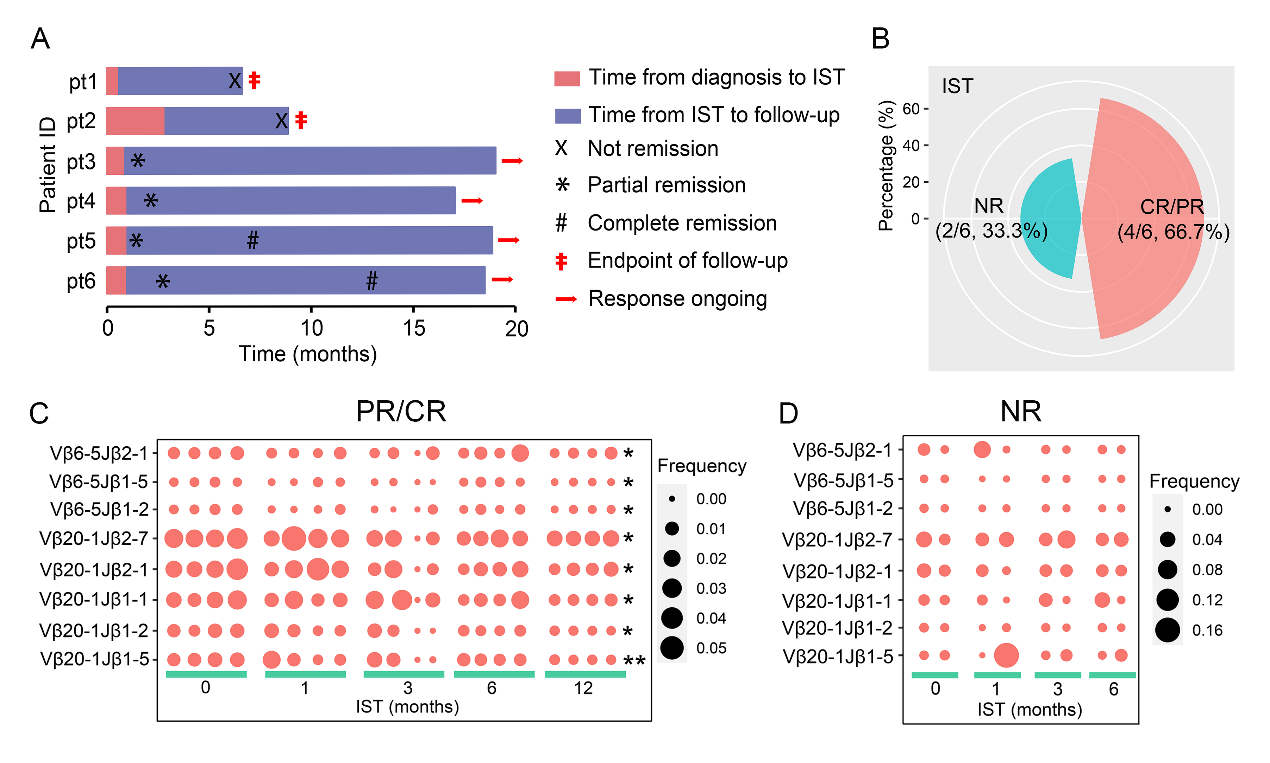


**Figure S4.** The difference in TCR rearrangement between the newly diagnosed and SAA patients receiving immunosuppressive therapy (IST) in the GZFPH dataset. (A) The follow-up of 6 SAA patients receiving IST. (B) Efficacy of SAA patients after receiving IST. C-D: Distribution characteristics of TCR rearrangements after SAA patients receiving IST in the partial remission (PR)/ complete remission (CR) and not remission (NR) subgroups. *, *P* < 0.05; **, *P* < 0.01.


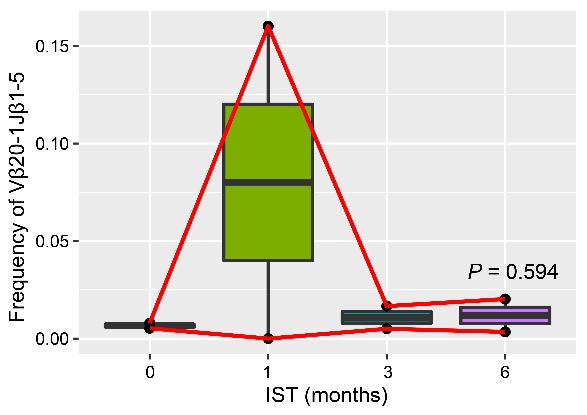


**Figure S5.** The frequency of Vβ20-1Jβ1-5 among SAA patients receiving IST for 0,1,3, and 6 months in the NR subgroup.


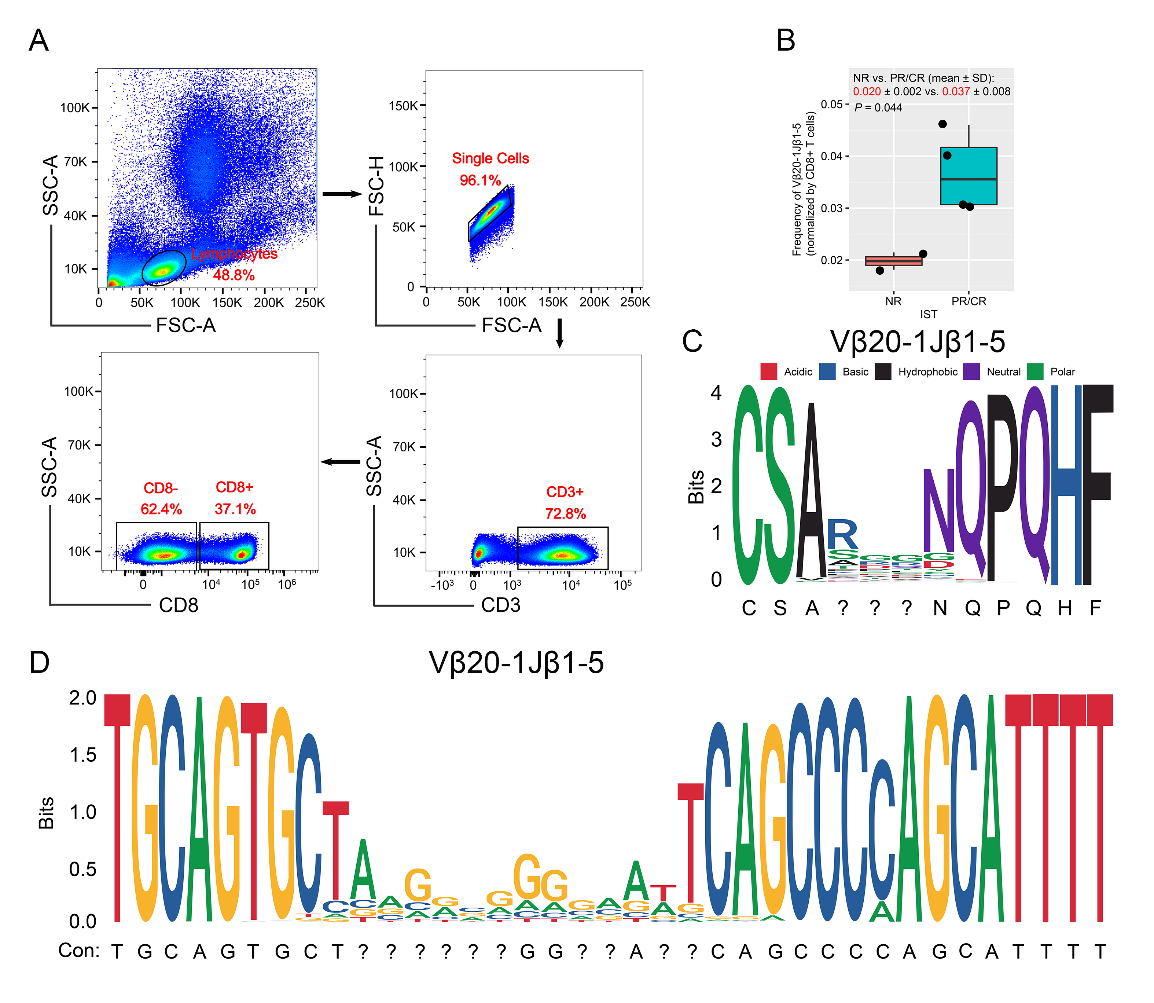


**Figure S6.** The frequency of Vβ20-1Jβ1-5 normalized by CD8+ T cells in the GZFPH dataset. (A) The logic plots of flow cytometry for detecting CD8+T cells. (B) The frequency of Vβ20-1Jβ1-5 normalized by CD8+ T cells between NR and PR/CR subgroups was compared. C-D: Identification of the conservative amino acid (C) and (D) nucleotide sequences of Vβ20-1Jβ1-5 by local alignment using the “msa” package. The question mark “?” indicates a non-conserved amino acid or nucleotide sequence at that position.

**Table S1.** Clinical characteristics of SAA patients.

| Variables | GZFPH | GSE101660 |
| --- | --- | --- |
| Total, n | 6 | 12 |
| Gender, n (%) |  |  |
| Female | 4 (66.7) | 5 (41.7) |
| Male | 2 (33.3) | 7 (58.3) |
| Age at IST, median, range, yrs | 29 (17 - 63) | 44 (14 - 71) |
| ECOG score |  | - |
| 0 | 1 (16.7) | - |
| 1 | 1 (16.7) | - |
| 2 | 4 (66.7) | - |

ECOG: Eastern Cooperative Oncology Group; IST: Immunosuppressive therapy.
